# Impact of Keratinized Mucosa Width and Mucosal Thickness on Implant Outcomes and Brushing Discomfort: A 25‐Year Cohort Study

**DOI:** 10.1111/jcpe.70187

**Published:** 2026-08-06

**Authors:** Emilio Couso‐Queiruga, Clemens Raabe, Andrea Roccuzzo, Giovanni E. Salvi, Manrique Fonseca, Vivianne Chappuis

**Affiliations:** ^1^ Department of Oral Surgery and Stomatology University of Bern School of Dental Medicine Bern Switzerland; ^2^ Shanghai Perio‐Implant Innovation Center, Institute of Integrated Oral, Craniofacial and Sensory Research, Shanghai Ninth People's Hospital Shanghai Jiao Tong University School of Medicine Shanghai China; ^3^ College of Stomatology, National Center for Stomatology, National Clinical Research Center for Oral Diseases, Shanghai Key Laboratory of Stomatology, Shanghai Research Institute of Stomatology Shanghai Jiao Tong University Shanghai China; ^4^ Department of Periodontology University of Bern School of Dental Medicine Bern Switzerland; ^5^ Department of Reconstructive Dentistry and Gerodontology University of Bern School of Dental Medicine Bern Switzerland

**Keywords:** clinical trial, dental implants, peri‐implantitis, phenotype, titanium

## Abstract

**Objective:**

To assess the impact of keratinized mucosa width (KMW) and mucosal thickness (MT) on peri‐implant conditions over 25 years in patients with adequate oral hygiene enrolled in regular supportive peri‐implant care.

**Methods:**

Partially edentulous patients rehabilitated with tissue‐level implants were clinically and radiographically evaluated by calibrated examiners. Patient‐reported brushing discomfort was recorded, and associations with variables of interest were analysed.

**Results:**

A total of 149 patients, 235 implants, with a mean follow‐up of 25.4 ± 3 years, were included. Peri‐implantitis prevalence was 11.1%, 0% and 7.1% in the 0‐mm, < 2‐mm and ≥ 2‐mm KMW groups, respectively (*p* = 0.98). Sites with 0 mm KMW showed significantly greater crestal bone loss (CBL) than < 2 mm (−0.28 mm) and ≥ 2 mm (−0.40 mm) (*p* = 0.01). Absence of KMW was associated with an increased risk of peri‐implant soft‐tissue dehiscence (PSTD) and implant platform visibility (*p* ≤ 0.01). No significant differences were observed for probing depth (PD), bleeding on probing (BOP), plaque index or peri‐implant diseases (*p* > 0.05). MT was not associated with CBL, PD, BOP, suppuration on probing (SOP) or peri‐implant diseases (*p* > 0.05). Thin MT (< 2 mm) was linked to increased PSTD (*p* = 0.005), greater implant platform visibility (*p* = 0.04) and lower plaque index (*p* = 0.01). Brushing discomfort was reported in 11.4% of sites with 0 mm KMW, compared with 3.4% and 2.4% in the < 2 mm and ≥ 2 mm groups, respectively, with no significant associations across KMW or MT groups (*p* > 0.05).

**Conclusions:**

KMW was associated with long‐term peri‐implant tissue stability, whereas thin MT was primarily associated with an increased risk of PSTD. Neither KMW nor MT was significantly associated with long‐term brushing discomfort.

## 1 Introduction

The *peri‐implant phenotype* refers to the morphological and dimensional characteristics that define the clinical presentation of the tissues surrounding and supporting osseointegrated dental implants (Avila‐Ortiz et al. 2020). The *peri‐implant keratinized mucosa width* (*KMW*) is defined as the vertical dimension of keratinized soft‐tissue extending in an apico‐coronal direction from the mucosal margin to the mucogingival junction and, when present, represents the most coronal aspect of the peri‐implant soft‐tissue phenotype (Avila‐Ortiz et al. 2020). *Peri‐implant mucosal thickness* (*MT*) refers to the horizontal dimension of the peri‐implant soft tissue, irrespective of whether it is keratinized or non‐keratinized. MT is particularly relevant for peri‐implant health (PH) and aesthetics in the coronal portion of the peri‐implant mucosa (Avila‐Ortiz et al. 2020).

Pre‐clinical (Warrer et al. 1995) and clinical studies (da Costa Deller et al. 2026; Isler et al. 2024; Lin et al. 2013; Perussolo et al. 2022; Ramanauskaite et al. 2022; Roos‐Jansåker et al. 2006; Warrer et al. 1995) evaluating the influence of KMW indicated higher plaque accumulation, increased bleeding on probing (BOP), peri‐implant soft‐tissue dehiscence (PSTD), brushing discomfort and crestal bone loss (CBL) at sites presenting inadequate KMW. The impact of limited KMW appears to be more pronounced in patients with irregular peri‐implant supportive care compliance (Monje and Blasi 2019). However, evidence regarding the relationship between KMW, implant‐related outcomes and peri‐implant mucositis (PM) or peri‐implantitis (PI) remains controversial, with some studies suggesting a potential association (Roccuzzo et al. 2025, 2023), while others report no significant relationship (Mancini et al. 2024), as reported in recent reviews (Ravidà et al. 2022; Sabri, Tavelli, et al. 2025).

With respect to MT, some studies have reported a higher prevalence of PSTD in the presence of thin MT (Isler et al. 2024; Tavelli et al. 2022). Conversely, sites presenting with thick MT, or those that underwent soft‐tissue phenotype modification procedures, have demonstrated improved clinical and radiographic stability (Tavelli et al. 2021), consistent with the higher PH rates reported following soft‐tissue augmentation procedures (Thoma et al. 2018). When PM or PI have been evaluated, the evidence appears similarly inconsistent to that reported for KMW. While some studies have reported no association between MT and PM or PI (Gao et al. 2024; Nícoli et al. 2024), others have suggested that greater MT may exert a protective effect (Couso‐Queiruga, Fonseca, Chappuis, et al. 2026; Sabri, Hazrati, et al. 2025).

Consequently, evidence evaluating the impact of KMW and MT in populations with adequate oral hygiene and regular peri‐implant supportive care remains limited, particularly in studies with long‐term follow‐up, as most available data are restricted to short‐ or medium‐term observations. Therefore, the primary aim of this 25‐year cohort study was to assess the influence of KMW on CBL. The secondary aims were (1) to evaluate the influence of KMW and MT on implant‐related outcomes (i.e., probing depth (PD), BOP, suppuration on probing (SOP), plaque index, PSTD) and peri‐implant conditions over time, and (2) to assess the association between KMW and MT and implant‐related outcomes, as well as patient‐reported discomfort during brushing.

## 2 Materials and Methods

### 2.1 Experimental Design, Centre and Ethical Approval

This study was designed as a cohort study and conducted at the Department of Oral Surgery and Stomatology, University of Bern School of Dental Medicine (Switzerland), in accordance with the principles of the 2018 Declaration of Helsinki and reported following the Strengthening the Reporting of Observational Studies in Epidemiology (STROBE) guidelines (von Elm et al. 2008). Because formal institutional ethics committee review was not yet routinely required for clinical research at the time the original cohort was assembled, the study protocol was approved (KEK‐BE‐No. 2023‐02279) and registered at ClinicalTrials.gov (NCT06599320) for the 25‐year follow‐up phase of this study as previously reported (Couso‐Queiruga, Roccuzzo, Raabe, et al. 2026).

### 2.2 Eligibility Criteria and Recruitment

All medical records of adult subjects who were included in a previously reported study (Buser et al. 2012) were reviewed to identify those eligible for re‐evaluation. Eligible patients were contacted and invited to undergo clinical and radiographic examination as part of the follow‐up phase.

All details regarding patient recruitment, as well as the inclusion and exclusion criteria, have been described previously (Raabe et al. 2026). Patients were excluded from the 25‐year follow‐up if they declined further comprehensive examinations or presented with disabilities or conditions impairing their ability to understand, read or sign the informed consent.

Similarly, only implant‐supported prostheses (ISPs) with available data at both the 10‐ and 25‐year follow‐ups were included. Sites lacking KMW measurements at either time point were excluded. Accordingly, the 10‐year component of this study is retrospective in nature, as these data were not collected prospectively for the present analysis; only the 25‐year examination was prospectively planned and conducted under the current study protocol.

### 2.3 Digital and Clinical Data Acquisition and Collection

At both time points, oral examinations were performed by calibrated periodontists following previously described protocols (Raabe et al. 2026). All implants were placed within the alveolar housing in a prosthetically driven, three‐dimensionally correct position, consistent with the placement protocol described in the original cohort (Buser et al. 2012).

Clinical parameters were recorded at six sites using a UNC‐15 periodontal probe and included PD, BOP, SOP, plaque index and KMW, which was measured at the mid‐facial aspect. MT was assessed only at the 25‐year follow‐up and measured at the mid‐facial aspect 3 mm apical to the mucosal margin using a standardized transgingival probing method, as previously described (Couso‐Queiruga, Fonseca, Rodrigues, et al. 2026; Couso‐Queiruga et al. 2021).

Standardized intraoral photographs of the region of interest were captured at both time points. One examiner (E.C‐Q.) evaluated the images for the presence of PSTD, visibility of implant platform or implant surface (Tavelli and Barootchi 2025) as shown in Figure 1. For PSTD assessment, a horizontal reference line indicating the ideal mucosal margin position was established. In central incisors and posterior sites, this reference was based on the marginal gingival levels of adjacent teeth. For lateral incisors and canines, the contralateral homologous tooth served as the reference; if absent, adjacent teeth were used. PSTD was defined as present when the mucosal margin was apical to the reference line and absent when it was at or coronal to it.

**FIGURE 1:**
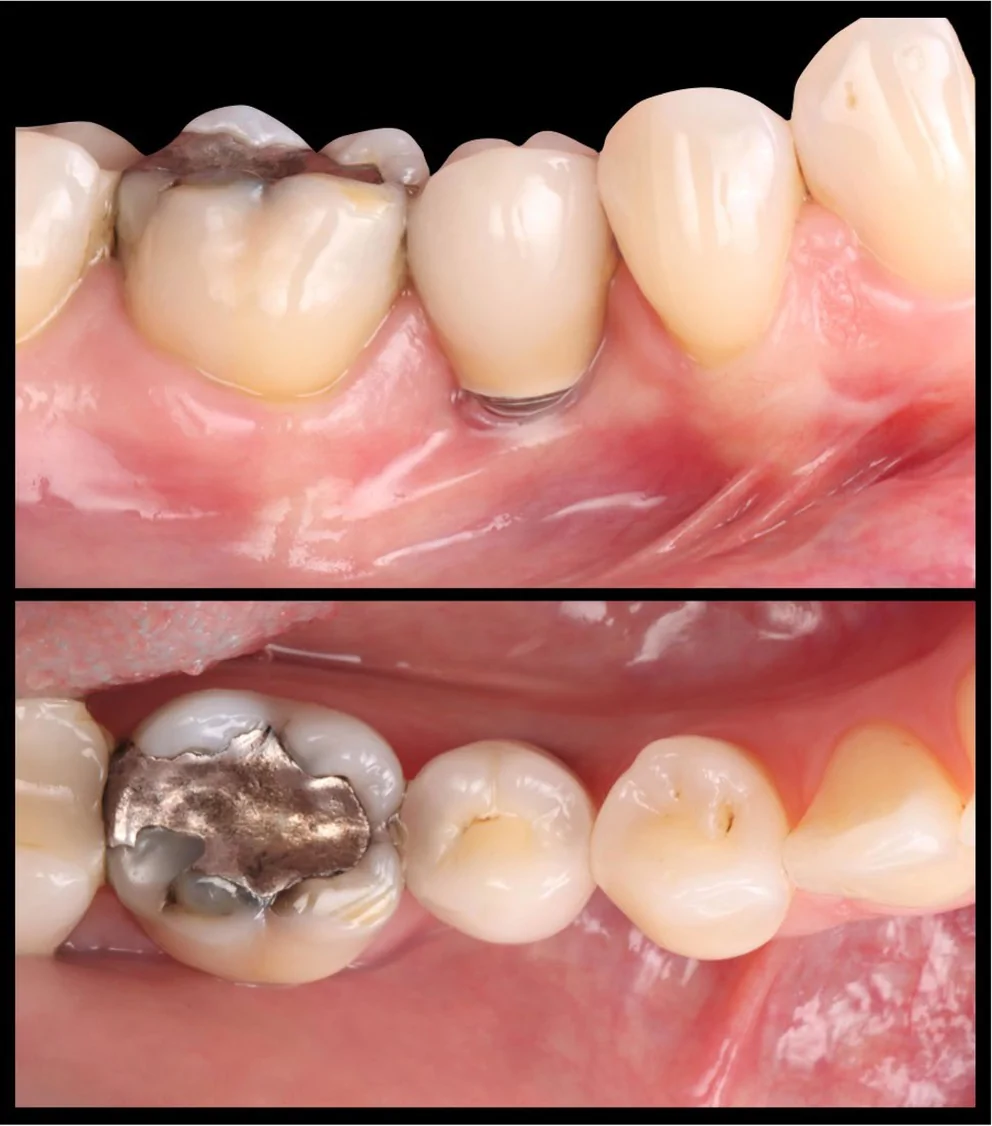
Clinical example of an implant‐supported prosthesis demonstrating peri‐implant mucosal dehiscence and implant platform visibility at a site with absent keratinized mucosa width and thin mucosal thickness at the 25‐year follow‐up.

Standardized digital periapical radiographs were obtained and evaluated as previously reported to assess CBL and ISP fit (Buser et al. 2012; Couso‐Queiruga, Ramseier, Chappuis, et al. 2026; Weber et al. 1992). Finally, patient demographics, medical and dental history, history of periodontitis, surgical and prosthetic variables, participation in supportive peri‐implant care and implant location were also recorded.

Peri‐implant conditions such as PH, PM and PI were defined based on the 2017 World Workshop Classification in Periodontal and Peri‐implant Diseases and Conditions (Renvert et al. 2018), and the European Federation of Periodontology Clinical Practice Guidelines (Herrera et al. 2023). These definitions were applied utilizing the baseline data.

### 2.4 Patient‐Reported Outcome Measures Related to Discomfort During Brushing

Before the clinical and radiographic evaluations at the 25‐year follow‐up, patients with multiple ISPs were asked to report brushing‐related discomfort for each ISP. Responses were recorded at the ISP level and classified dichotomously as “yes” or “no”.

Further details related to the statistical analysis are available in the Data S1.

## 3 Results

### 3.1 Patient Sample Characteristics

A total of 149 patients (84 women [56.4%] and 65 men [43.6%]) with a mean age of 72.2 ± 13.2 years met the inclusion criteria, yielding a total of 235 ISPs available for analysis at both time points (10 and 25 years). Brushing discomfort was observed in 11.4% of ISPs in the 0‐mm KMW group, compared with 3.4% and 2.4% in the < 2‐mm and ≥ 2‐mm groups, respectively, without significant differences between KMW or MT groups (*p* > 0.05). The mean follow‐up period was 25.4 ± 3.0 years.

Detailed demographic and site‐specific characteristics are presented in Table 1.

**TABLE 1:** Demographic and sample characteristics according to the keratinized mucosa width stratification.

Characteristic	0 mm KMW *N* = 36	< 2 mm KMW *N* = 29	≥ 2 mm KMW *N* = 170	*p*
Gender				0.77
Male	17 (47.2%)	11 (37.9%)	79 (46.5%)	
Female	19 (52.8%)	18 (62.1%)	91 (53.5%)	
Age (years)	77 [78] (9)	71 [75] (15)	72 [77] (14)	0.13
Smoking status				0.65
No	32 (88.9%)	27 (93.1%)	148 (87.1%)	
Yes/Former smoker	4 (11.1%)	2 (6.9%)	22 (12.9%)	
Implant diameter				0.37
3.3 mm	0 (0.0%)	0 (0.0%)	8 (4.7%)	
4.1 mm	14 (38.9%)	15 (51.7%)	94 (55.3%)	
4.8 mm	22 (61.1%)	14 (48.3%)	68 (40.0%)	
Implant length				0.06
≤ 10 mm	30 (83.3%)	18 (62.1%)	103 (60.6%)	
> 12 mm	6 (16.7%)	11 (37.9%)	67 (39.4%)	
Platform				1.00
4.8 mm	31 (86.1%)	25 (86.2%)	135 (79.4%)	
6.5 mm	5 (13.9%)	4 (13.8%)	27 (15.9%)	
3.5 mm	0 (0.0%)	0 (0.0%)	8 (4.7%)	
Number of implants				0.14
Single implant	2 (5.6%)	8 (27.6%)	36 (21.2%)	
Multiple implants	34 (94.4%)	21 (72.4%)	134 (78.8%)	
Reconstruction				0.59
Single crown	14 (38.9%)	16 (55.2%)	99 (58.2%)	
Splinted crown	10 (27.8%)	8 (27.6%)	43 (25.3%)	
Bridges and removable attachments	12 (33.3%)	5 (17.2%)	28 (16.5%)	
Location				0.59
Mandibula	21 (58.3%)	20 (69.0%)	98 (57.6%)	
Maxilla	15 (41.7%)	9 (31.0%)	72 (42.4%)	
Position				0.96
Incisors	0 (0.0%)	0 (0.0%)	20 (11.8%)	
Canines	2 (5.6%)	3 (10.3%)	13 (7.6%)	
Premolars	14 (38.9%)	14 (48.3%)	69 (40.6%)	
Molars	20 (55.6%)	12 (41.4%)	68 (40.0%)	
Bone augmentation procedures				0.87
None	25 (69.4%)	21 (72.4%)	124 (72.9%)	
Guided bone regeneration	5 (13.9%)	2 (6.9%)	31 (18.2%)	
Sinus floor elevation	6 (16.7%)	6 (20.7%)	15 (8.8%)	
Bone augmentation timing				0.98
Simultaneous	7 (19.4%)	3 (10.3%)	26 (15.3%)	
Staged	4 (11.1%)	5 (17.2%)	20 (11.8%)	
Retention				0.55
Cement retained	32 (88.9%)	24 (82.8%)	132 (77.6%)	
Screw retained	4 (11.1%)	5 (17.2%)	38 (22.4%)	
Fit of reconstruction				< 0.001*
Good	33 (91.7%)	27 (93.1%)	142 (83.5%)	
Questionable	3 (8.3%)	1 (3.4%)	27 (15.9%)	
Poor	0 (0.0%)	1 (3.4%)	1 (0.6%)	
Mucosal thickness				0.95
< 2 mm	15 (41.7%)	11 (39.3%)	60 (36.8%)	
≥ 2 mm	21 (58.3%)	17 (60.7%)	103 (63.2%)	

*Note: n* (%); Mean [Median]; (SD).

Abbreviations: KMW, keratinized mucosa width; mm, millimetres.

* Statistical significance.

### 3.2 Variables of Interest Stratified by Keratinized Mucosa Width

At 10 and 25 years, statistically significantly greater CBL was observed in ISPs with 0 mm KMW (0.29 ± 0.79 mm and 0.62 ± 1.04 mm, respectively) compared with ISPs presenting < 2 mm (−0.18 ± 0.80 mm and 0.01 ± 0.77 mm) and ≥ 2 mm KMW (−0.18 ± 0.81 mm and 0.24 ± 1.26 mm) (*p* = 0.01). PD values (10 years, *p* = 0.04; 25 years, *p* = 0.07), as well as cumulative BOP and SOP, were similar among groups (*p* > 0.05) and over time.

At the 10‐year follow‐up, PI was observed in 2 ISPs in the 0‐mm group (5.5%) and in 2 ISPs in the ≥ 2‐mm group (1.2%), while no cases were detected in the < 2‐mm group (0.0%) (*p* = 0.58). PM was the most prevalent condition across all groups, affecting 27 (75.0%), 24 (82.8%) and 140 (82.4%) ISPs in the 0‐mm, < 2‐mm and ≥ 2‐mm groups, respectively (*p* = 1.00). At the 25‐year follow‐up, the prevalence of PI was 4 cases (11.1%) in the 0‐mm group, and 0 in the < 2‐mm group (0.0%), and was observed in 12 cases (7.1%) in the ≥ 2‐mm group (*p* = 0.07). PM was the predominant diagnosis, affecting 29 (80.6%), 27 (93.1%) and 128 (75.3%) ISPs in the 0‐mm, < 2‐mm and ≥ 2‐mm groups, respectively (*p* = 0.98).

PSTD was observed in 97.2% of ISPs in the 0‐mm KMW group at both time points. In the < 2‐mm group, it was 69.9% and 75.9% at the 10‐ and 25‐year assessments, respectively, while in the ≥ 2‐mm group it was 54.7% and 61.8% at the respective assessments. At both time points, the 0‐mm KMW group exhibited a statistically significantly higher prevalence of PSTD (*p* < 0.05). Similarly, visibility of implant platform was most frequent in the 0‐mm group (58.3% and 86.1%), followed by the < 2 mm (51.7% and 62.1%) and ≥ 2‐mm groups (17.1% and 36.5%) (*p* < 0.01) at 10 and 25 years, respectively. No endosseous implant surface exposure was observed at any time point.

Clinical and radiographic findings stratified according to the KMW values are summarized in Table 2, while corresponding examples are presented in Figure 2.

**TABLE 2:** Variables of interest according to the keratinized mucosa width stratification.

Variables of interest	0 mm KMW *N* = 36	< 2 mm KMW *N* = 29	≥ 2 mm KMW *N* = 170	*p*
10 years follow‐up				
Mean crestal bone levels (mm)	0.29 [0.17] (0.79)	−0.18 [−0.21] (0.80)	−0.18 [−0.31] (0.81)	0.01*
Mean probing depths (mm)	3.12 [3.08] (0.86)	2.95 [2.83] (0.74)	3.32 [3.25] (0.80)	0.04*
Mean bleeding on probing	2.81 [2.50] (1.51)	3.31 [3.00] (1.81)	3.32 [3.00] (1.70)	0.92
Plaque index	3.33 [3.00] (2.14)	3.28 [3.00] (2.02)	3.02 [3.00] (2.05)	0.58
Mean suppuration on probing				1.00
No	35 (97.2%)	29 (100.0%)	170 (100.0%)	
Yes	1 (2.8%)	0 (0.0%)	0 (0.0%)	
Peri‐implant conditions				1.00
Peri‐implantitis	2 (5.6%)	0 (0.0%)	2 (1.2%)	
Peri‐implant mucositis	27 (75.0%)	24 (82.8%)	140 (82.4%)	
Peri‐implant health	7 (19.4%)	5 (17.2%)	28 (16.5%)	
Peri‐implant mucosal dehiscence				< 0.001*
No	1 (2.8%)	9 (31.0%)	77 (45.3%)	
Yes	35 (97.2%)	20 (69.0%)	93 (54.7%)	
Visibility of implant platform				< 0.001*
No	15 (41.7%)	14 (48.3%)	141 (82.9%)	
Yes	21 (58.3%)	15 (51.7%)	29 (17.1%)	
Visibility of implant surface				—
No	36 (100.0%)	29 (100.0%)	170 (100.0%)	
25 years follow‐up				
Mean crestal bone levels (mm)	0.62 [0.43] (1.04)	0.01 [0.05] (0.77)	0.24 [0.00] (1.26)	0.01*
Mean probing depths (mm)	2.82 [2.50] (1.45)	2.81 [2.67] (0.87)	3.10 [3.00] (1.08)	0.07
Mean bleeding on probing	2.67 [2.00] (1.41)	2.45 [2.00] (1.02)	2.52 [2.00] (1.47)	0.56
Plaque index	0.31 [0.00] (1.19)	0.48 [0.00] (0.95)	0.48 [0.00] (1.02)	0.07
Mean suppuration on probing				0.82
No	34 (94.4%)	29 (100.0%)	164 (96.5%)	
Yes	2 (5.6%)	0 (0.0%)	6 (3.5%)	
Peri‐implant conditions				0.98
Peri‐implantitis	4 (11.1%)	0 (0.0%)	12 (7.1%)	
Peri‐implant mucositis	29 (80.6%)	27 (93.1%)	128 (75.3%)	
Peri‐implant health	3 (8.3%)	2 (6.9%)	30 (17.6%)	
Peri‐implant mucosal dehiscence				0.006*
No	1 (2.8%)	7 (24.1%)	65 (38.2%)	
Yes	35 (97.2%)	22 (75.9%)	105 (61.8%)	
Visibility of implant platform				< 0.001*
No	5 (13.9%)	11 (37.9%)	108 (63.5%)	
Yes	31 (86.1%)	18 (62.1%)	62 (36.5%)	
Visibility of implant surface				—
No	36 (100.0%)	29 (100.0%)	170 (100.0%)	

*Note: n* (%). Mean [Median]; (SD).

Abbreviations: KMW, keratinized mucosa width; mm, millimetres.

* Statistical significance.

**FIGURE 2:**
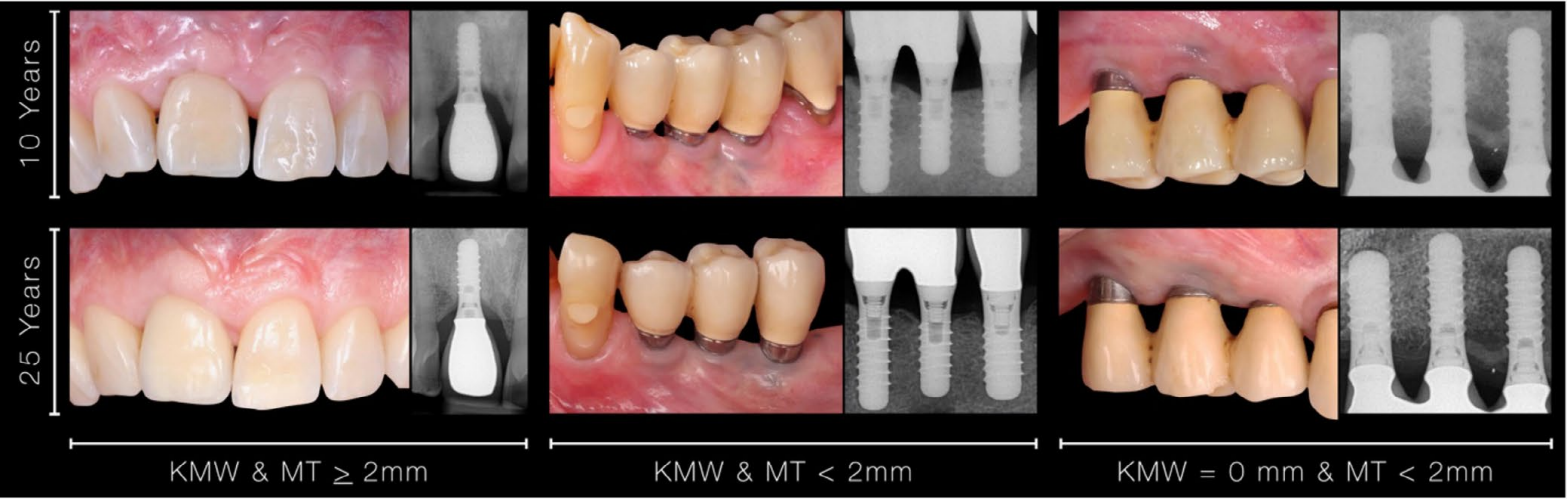
Clinical and radiographic images stratified by keratinized mucosa width (KMW), with corresponding mucosal thickness (MT) values at the 10‐ and 25‐year follow‐ups.

### 3.3 Associations Between Keratinized Mucosa Width and Variables of Interest

Univariate analyses at 10 years revealed no significant differences between groups in PI (*p* = 0.40 and *p* = 0.06) nor the presence of SOP at either time point (*p* = 1.00 and *p* = 0.82). No statistically significant differences were observed in the prevalence of PI, PM or PH across KMW stratifications at 10 years (*p* = 0.98) or 25 years (*p* = 0.99).

At 10 years, ISPs with 0 mm KMW showed significantly greater mean CBL compared to ISPs with < 2 mm and ≥ 2 mm KMW (< 2 mm: −0.41 mm [95% confidence interval, CI: −0.74, −0.08]; ≥ 2 mm: −0.43 mm [−0.70, −0.16]; *p* = 0.01). A comparable association was observed at 25 years (< 2 mm: −0.25 mm [−0.58, 0.07]; ≥ 2 mm: −0.40 mm [−0.67, −0.13]; *p* = 0.01).

Higher mean PD values were generally associated with ≥ 2 mm KMW, achieving statistical significance only at the 10‐year follow‐up (0.15 mm [95% CI: −0.05, 0.34], *p* = 0.04). Mean cumulative BOP did not differ significantly across KMW groups at either follow‐up (*p* = 0.92 and *p* = 0.56), as well as plaque (*p* = 0.58 and *p* = 0.07).

ISPs with reduced KMW exhibited a significantly higher risk of PSTD and greater visibility of the implant platform. At 10 years, < 2‐mm and ≥ 2‐mm ISPs had lower odds of PSTD (< 2 mm: 0.10 [95% CI: 0.02, 0.47]; ≥ 2 mm: 0.07 [95% CI: 0.02, 0.27]; *p* < 0.001) and visibility of implant platform (< 2 mm: 0.76 [95% CI: 0.33, 1.72]; ≥ 2 mm: 0.20 [95% CI: 0.11, 0.39]; *p* < 0.001) compared with 0 mm ISPs. Comparable associations were noted at 25 years for both PSTD (< 2 mm: 0.24 [95% CI: 0.06, 0.89]; ≥ 2 mm: 0.17 [95% CI: 0.05, 0.51]; *p* < 0.05) and visibility of implant platform (< 2 mm: 0.29 [95% CI: 0.09, 0.93]; ≥ 2 mm: 0.14 [95% CI: 0.06, 0.38]; *p* < 0.001) as presented in Table 3.

**TABLE 3:** Multivariate analyses between keratinized mucosa width stratification and clinical and other variables of interest, presented as effect estimates with 95% confidence intervals.

Variables of interest	Keratinized mucosa width at 10 years		*p*	Keratinized mucosa width at 25 years		*p*
Mean crestal bone levels (mm)	0 mm < 2 mm ≥ 2 mm	Baseline −0.41 [−0.74, −0.08] −0.43 [−0.70–0.16]	0.01*	0 mm < 2 mm ≥ 2 mm	Baseline −0.25 [−0.58, 0.07] −0.40 [−0.67, −0.13]	0.01*
Mean probing depths (mm)	0 mm < 2 mm ≥ 2 mm	Baseline −0.10 [−0.34, 0.14] +0.15 [−0.05, 0.34]	0.04*	0 mm < 2 mm ≥ 2 mm	Baseline −0.13 [−0.43, 0.18] +0.17 [−0.09, 0.43]	0.07
Mean bleeding on probing	0 mm < 2 mm ≥ 2 mm	Baseline −0.10 [−0.97, 0.76] +0.04 [−0.66, 0.70]	0.92	0 mm < 2 mm ≥ 2 mm	Baseline +0.10 [−0.50, 0.71] +0.25 [−0.23, 0.74]	0.56
Plaque index	0 mm < 2 mm ≥ 2 mm	Baseline −0.10 [−0.88, 0.67] −0.32 [−0.96, 0.33]	0.58	0 mm < 2 mm ≥ 2 mm	Baseline +0.13 [−0.02, 0.28] +0.13 [0.02, 0.24]	0.07
Peri‐implant mucosal dehiscence	0 mm < 2 mm ≥ 2 mm	Baseline 0.10 [0.02, 0.47] 0.07 [0.02, 0.27]	< 0.001*	0 mm < 2 mm ≥ 2 mm	Baseline 0.24 [0.06, 0.89] 0.17 [0.05, 0.51]	0.006*
Visibility of implant platform	0 mm < 2 mm ≥ 2 mm	Baseline 0.76 [0.33, 1.72] 0.20 [0.11, 0.39]	< 0.001*	0 mm < 2 mm ≥ 2 mm	Baseline 0.29 [0.09, 0.93] 0.14 [0.06, 0.38]	< 0.001*

Abbreviation: mm, millimetres.

* Statistical significance.

### 3.4 Associations Between Mucosal Thickness and Variables of Interest

Univariate analyses demonstrated that MT was not statistically associated with SOP (25 years: *p* = 0.59; 10‐year data did not converge) or with PI or PM (10 years: *p* = 1.00; 25 years: *p* = 0.99).

Although a lower mean CBL was observed at 10 (−0.13 mm; *p* = 0.17) and 25 years (−0.06 mm; *p* = 0.56) in the thick (≥ 2 mm) MT group, the differences were not significant between thin (< 2 mm) and thick mucosa phenotypes. Similarly, no significant differences were noted for the mean PD values (10 years: 0.07, *p* = 0.34, 25 years: +0.01 mm; *p* = 0.88), or cumulative BOP (10 years: +0.16; *p* = 0.51, 25 years: +0.12 mm; *p* = 0.51).

Contrarily, thicker MT was associated with higher plaque (10 years: +0.30; *p* = 0.21, 25 years: +0.12; *p* = 0.01), and statistically significantly lower risk of PSTD (10 years: 0.46; *p* = 0.01, 25 years: 0.37; *p* = 0.005) and visibility of implant platform (10 years: 0.67; *p* = 0.40, 25 years: 0.51; *p* = 0.04) as presented in Table 4.

**TABLE 4:** Multivariate analyses between mucosal thickness stratification and variables of interest, presented as effect estimates with 95% confidence intervals.

Variables of interest	Mucosal thickness at 10 years		*p*	Mucosal thickness at 25 years		*p*
Mean crestal bone levels (mm)	< 2 mm ≥ 2 mm	Baseline −0.13 [−0.32, 0.06]	0.17	< 2 mm ≥ 2 mm	Baseline −0.06 [−0.25, 0.13]	0.56
Mean probing depths (mm)	< 2 mm ≥ 2 mm	Baseline +0.07 [−0.07, 0.21]	0.34	< 2 mm ≥ 2 mm	Baseline +0.01 [−0.17, 0.20]	0.88
Mean bleeding on probing	< 2 mm ≥ 2 mm	Baseline +0.16 [−0.32, 0.65]	0.51	< 2 mm ≥ 2 mm	Baseline +0.12 [−0.23, 0.46]	0.51
Plaque index	< 2 mm ≥ 2 mm	Baseline +0.30 [−0.17, 0.76]	0.21	< 2 mm ≥ 2 mm	Baseline +0.12 [0.03, 0.20]	0.01*
Peri‐implant mucosal dehiscence	< 2 mm ≥ 2 mm	Baseline 0.46 [0.25, 0.84]	0.01*	< 2 mm ≥ 2 mm	Baseline 0.37 [0.19, 0.70]	0.005*
Visibility of implant platform	< 2 mm ≥ 2 mm	Baseline 0.67 [0.34, 1.34]	0.40	< 2 mm ≥ 2 mm	Baseline 0.51 [0.29, 0.89]	0.04*

Abbreviation: mm, millimetres.

* Statistical significance.

### 3.5 Associations of Keratinized Mucosa Width and Mucosal Thickness on Brushing Discomfort

Univariate analyses demonstrated that brushing discomfort did not differ significantly among the KMW groups (*p* = 0.27). Similarly, comparisons across the MT groups revealed no significant differences in brushing discomfort (*p* = 0.83).

## 4 Discussion

### 4.1 Main Findings

This cohort study evaluated the 25‐year impact of KMW and MT on clinical and radiographic implant outcomes, peri‐implant conditions and brushing discomfort in patients generally enrolled in supportive peri‐implant care and presenting adequate oral hygiene. Although slight differences in clinical and radiographic parameters and peri‐implant conditions were observed across KMW and MT groups, multivariate analyses showed that the absence of KMW was significantly associated with greater CBL, higher prevalence of PSTD and increased implant platform visibility. Thin MT (< 2 mm) was associated with lower plaque values, but also with higher prevalence of PSTD and implant platform visibility. Neither KMW nor MT was associated with long‐term brushing discomfort or PI prevalence. Because MT was assessed only at the single 25‐year examination, its associations with concurrent and earlier outcomes reported below are necessarily cross‐sectional; a temporally ordered exposure–outcome relationship cannot be established, and the possibility of reverse causation cannot be excluded.

### 4.2 Agreements and Disagreements With Existing Evidence

ISPs with 0 mm KMW exhibited significantly higher CBL at both timepoints, whereas ISPs with ≥ 2 mm KMW showed the lowest CBL. A similar trend was observed when comparing MT phenotypes, although the differences were not statistically significant. These results align with those of systematic reviews reporting that ISPs with inadequate KMW or ISPs with thin MT that did not undergo phenotype modification therapies demonstrate higher CBL compared to ISPs with adequate KMW or thick MT (Carra et al. 2023; Tavelli et al. 2021). In the present cohort, mean PD values did not differ significantly between KMW and MT groups, consistent with previous reports (Carra et al. 2023; da Costa Deller et al. 2026). Likewise, no significant differences were observed in BOP, plaque index or SOP between groups, except for plaque index in the MT phenotype groups. Although plaque was more frequent in the 0‐mm KMW group, no statistically significant associations were observed between either KMW or MT and plaque, as reported in other studies (Lim et al. 2019; Oliveira et al. 2024). In contrast, prior studies reported that patients with inadequate KMW and irregular supportive peri‐implant care experience higher CBL and increased peri‐implant disease rates (Monje and Blasi 2019; Roccuzzo et al. 2025). The present findings may be explained by characteristics of this Swiss cohort, demonstrating good oral hygiene habits, regular compliance and the absence of additional risk factors. These conditions suggest that KMW may not be a decisive local factor in the onset of PI, as also reported in other studies (da Costa Deller et al. 2026; Ravidà et al. 2022; Sabri, Tavelli, et al. 2025). Regarding MT, studies also report no association with peri‐implant diseases (Gao et al. 2024; Isler et al. 2024; Nícoli et al. 2024). However, a recent study from our group observed that thicker MT was associated with lower odds of PM and PI in non‐molar sites (Couso‐Queiruga, Fonseca, Chappuis, et al. 2026), consistent with another study showing reduced PM in ISPs with MT > 1.5 mm (Sabri, Hazrati, et al. 2025). Our findings may be explained by the stratification of MT, rather than its evaluation as a continuous variable, which may have resulted in a spurious interaction (Couso‐Queiruga, Roccuzzo, Raabe, et al. 2026; Thoresen 2019).

When evaluating PSTD, ISPs lacking KMW, followed by those with < 2 mm KMW, or presenting thin MT, exhibited the highest prevalence of PSTD and visibility of implant platform, compared to ISPs with ≥ 2 mm KMW or thick MT. Our prevalence of PSTD was higher, with platform visibility being relatively similar in the 0 mm and < 2‐mm KMW groups as reported in previous studies over shorter follow‐up periods (Couso‐Queiruga, Leoncini, Fonseca, et al. 2026; Tavelli and Barootchi 2025). Nevertheless, in the present cohort, PI was also included in the analyses, and the follow‐up was substantially longer. Previous reports have reported an association between longer follow‐up and a higher prevalence of PSTDs (Sanz‐Martín et al. 2022; Tavelli and Barootchi 2025; Tavelli et al. 2022). An exception was observed in the 0‐mm KMW group, where a 97.2% prevalence of PSTDs was noted at the 10‐ and 25‐year follow‐ups, with only one ISP not developing PSTD. These findings align with previous studies reporting greater PSTD in ISPs with absent KMW (Crespi et al. 2019; da Costa Deller et al. 2026; Isler et al. 2024; Roccuzzo et al. 2025), and thin MT (Isler et al. 2024; Nisapakultorn et al. 2010; Tavelli et al. 2022). Additionally, the present findings may have been influenced by factors such as implant type and platform diameter, ISP, implant position and the extended follow‐up period, among others (Chambrone et al. 2026; Couso‐Queiruga and Chambrone 2026; Couso‐Queiruga, Raabe, Rubio‐Ferrer, et al. 2026; Moser et al. 2026). Nevertheless, no implants in this cohort were considered malpositioned.

Finally, neither KMW nor MT showed significant associations with brushing discomfort, although the 0‐mm KMW group had the highest frequency. This contrasts with shorter‐term studies (Gharpure et al. 2021; Monje and Blasi 2019; Perussolo et al. 2022; Souza et al. 2016), but aligns with others (Bonino et al. 2018; da Costa Deller et al. 2026; Ueno et al. 2016). Notably, a prior 10‐year evaluation of this same cohort found KMW < 2 mm was associated with increased discomfort (da Costa Deller et al. 2026). To our knowledge, this is the first study to evaluate MT and brushing discomfort over 25 years, finding no significant association, differing from a shorter‐term study (Gharpure et al. 2021), that stratified MT using probe visibility, a method recently shown to lack accuracy (Couso‐Queiruga, Fonseca, Rodrigues, et al. 2026; Moreira Rodrigues et al. 2026). Discrepancies across studies may also reflect differences in toothbrush type, brushing force, pain threshold and reliance on self‐report, since discomfort may itself lead patients to avoid brushing affected sites, biasing results; indeed, brushing discomfort may reflect peri‐implant disease rather than insufficient soft‐tissue dimensions alone (Perussolo et al. 2022). The absence of a long‐term association may also reflect adaptive biological mechanisms, including reduced neuronal activation with repeated stimulation (Klingner et al. 2014), site‐specific habituation (Jepma et al. 2014), nociceptor plasticity (Pace et al. 2018), altered oral mucosal somatosensory function as seen in gingivitis (Wang et al. 2020), neuroplasticity from sustained non‐harmful stimulation (McCarberg and Peppin 2019) and interindividual phenotypic variability (Harrison et al. 2025).

Further details related to the limitations of this study are available in Data S1.

## 5 Conclusions

Within the limitations of this 25‐year cohort study, with generally adequate oral hygiene and regular supportive peri‐implant care, the following conclusions can be drawn:

- The absence of KMW was associated with greater CBL, as well as an increased risk of PSTD and visibility of implant platform as compared to inadequate (< 2 mm) or adequate (≥ 2 mm) KMW.
- Thick (≥ 2 mm) MT was associated with a reduced risk of PSTD and visibility of implant platform.
- No statistically significant association was observed between brushing discomfort and the presence or absence of KMW or MT.
- No statistically significant association was detected between KMW and MT with PM or PI.

## Author Contributions

E.C.‐Q. conceived and designed the idea. E.C.‐Q., C.R., A.R., G.E.S. and M.F. contributed to clinical and digital data collection. E.C.‐Q. and C.R. contributed to digital data analysis. E.C.‐Q. led the writing. C.R., A.R., G.E.S., M.F. and V.C. contributed to data interpretation and critically revised the manuscript. All authors approved the final version and agreed to be responsible for all aspects of the work.

## Funding

This project was supported by the International Team for Implantology (project ID: 1574‐2021). Financial and material support was provided by the Departments of Oral Surgery and Stomatology, Periodontology, and Reconstructive Dentistry and Gerodontology, School of Dental Medicine, University of Bern.

## Conflicts of Interest

The authors declare no conflicts of interest.

## Supporting information


**Data S1:** Supporting Information.

## Data Availability

The data that support the findings of this study are available from the corresponding author upon reasonable request.
