# Supplementary material for: Impact of Keratinized Mucosa Width and Mucosal Thickness on Implant Outcomes and Brushing Discomfort: A 25‐Year Cohort Study
Source: J Clin Periodontol. 2026 Aug 6;53(11):1691–701. doi: 10.1111/jcpe.70187 (PMC13658043; doi:10.1111/jcpe.70187)
Supplement: Supplementary file 1 [file JCPE-53-1691-s001.docx]

**2.5. Statistical Analyses**

All analyses were conducted using R, version 4.5.2, with the packages **tidyverse**, **robustlmm**, **multpois**, **geepack**, **gtsummary**, and **DescTools**. Demographic, implant-, and prosthetic-related characteristics, as well as study outcomes, were stratified by the three keratinized mucosa width (KMW) groups. Continuous variables were summarized as means, medians, and standard deviations, while categorical variables were presented as frequencies and percentages. The implant-supported prosthesis (ISP) was considered the primary unit of analysis. Because some patients contributed more than one ISP, all inferential models accounted for within-patient clustering by including the patient as a clustering factor. Other clinical parameters were recorded at six sites but summarized at the ISP level for the purposes of the KMW/MT analyses. 'Patient' is used only when referring to patient-level demographic or clustering variables

The primary endpoint was the association between KMW and crestal bone loss. All other comparisons, including mucosal thickness associations, probing depth, bleeding on probing, suppuration on probing, plaque, peri-implantitis/peri-implant mucositis/peri-implant health prevalence, peri-implant soft tissue dehiscences, implant platform visibility, and brushing discomfort, for both KMW and MT were considered secondary or exploratory and were not adjusted for multiplicity; p-values for these analyses should be interpreted as descriptive/hypothesis-generating rather than confirmatory

Differences between KMW groups were evaluated using robust linear mixed-effects models for continuous outcomes, as standard mixed models were sensitive to outliers and deviations from normality. Binary outcomes were analyzed using generalized estimating equation (GEE) models, and nominal categorical outcomes were assessed using multinomial-Poisson generalized linear mixed models. In all models, the patient was included as a clustering factor to account for multiple reconstructions contributed by the same individual. Model fit was assessed through inspection of residuals.

Associations of mucosa thickness (MT) stratified as thin (<2 mm), and thick (≥2 mm), and KMW (0 mm, <2 mm, and ≥2 mm) with continuous outcomes were examined using robust linear mixed-effects models. For binary outcomes, GEE models were employed, with potentially relevant covariates (identified from descriptive comparisons with p < 0.1) considered for inclusion alongside MT and KMW. To prevent overparameterization, multivariable binary-outcome models were fitted only when the ratio of the smaller of events or non-events per model parameter exceeded five; otherwise, MT and KMW were modeled separately. Goodness-of-fit for GEE models was evaluated using the Hosmer–Lemeshow test. When parameters showed significant associations, post hoc pairwise comparisons were performed with Bonferroni–Holm adjustment.

Covariates were screened using a data-driven approach (univariable p<0.1) due to the exploratory nature of the secondary analyses and the limited number of events for several outcomes, which constrained the number of covariates that could be reliably estimated. We acknowledge that a priori covariate selection based on clinical knowledge or a causal framework (e.g., a directed acyclic graph) is generally preferable and reduces the risk of overfitting and collider bias; the data-driven approach used here should therefore be considered a pragmatic constraint of sample size rather than a methodological preference, and findings from these adjusted models should be interpreted as exploratory.

For outcomes with sparse events or zero-cell counts (e.g., peri-implantitis in the < 2 mm KMW group, suppuration on probing at 10 years), standard GEE/logistic estimates may be unstable or fail to converge; where convergence issues occurred, this is noted explicitly, and results for these outcomes should be interpreted with caution. Given these constraints, effect estimates and their 95% confidence intervals are emphasized over p-values alone.

Statistical significance was defined as a two-sided p-value ≤ 0.05.

**2.6. Sample Size Calculation**

A post-hoc sample size calculation was performed using effect sizes from a previously published, independent study, which reported marginal bone levels of 0.66 ± 0.70 mm in sites with KMW versus 1.29 ± 0.99 mm in sites without KMW (Roccuzzo et al., 2025), corresponding to Cohen's d = 0.735. Because the observed KMW groups in this cohort were unequal in size (0 mm: n=36; <2 mm: n=29; ≥2 mm: n=170) and sites were clustered within patients (149 patients contributing 235 sites; mean 1.58 sites/patient), achieved power was recalculated for each pairwise comparison using the observed sample sizes, with a design-effect correction for clustering across a range of plausible intraclass correlation coefficients (ICC 0.01–0.20). Because of the small average cluster size, the clustering correction had a minimal effect on power (design effect 1.01–1.12). Achieved power for the primary crestal bone levels comparisons was 82.6% (0mm vs <2mm), 97.9% (0mm vs ≥2mm), and 95.3% (<2mm vs ≥2mm) without clustering adjustment, and 80.4–81.5%, 97.2–97.6%, and 94.3–94.8%, respectively, under ICC assumptions of 0.05–0.10. The comparison between the two smallest groups (0mm vs <2mm) was therefore modestly underpowered relative to the conventional 80–95% threshold, and this should be considered when interpreting non-significant findings involving these two groups

**4.3. Limitations**

Despite adhering to high methodological standards, this study is not exempt from limitations. First, its cohort design precludes causal inference, and the reported associations between KMW, MT, and implant-related outcomes may be influenced by residual confounding during the long follow-up period (e.g., variations in oral hygiene practices and peri-implant supportive therapy compliance). Second, only tissue-level titanium implants were included, and the cohort consisted of a Swiss population enrolled in regular supportive peri-implant care with generally adequate oral hygiene. Therefore, the findings may not be fully generalizable to other populations, implant systems, or patients with less stringent maintenance protocols and oral hygiene habits. Third, the inclusion of only ISPs present at both the 10- and 25-year follow-ups introduces potential survivorship bias, as implant failures were not captured. Fourth, MT was assessed only at the 25-year follow-up, precluding the evaluation of temporal associations and limiting the ability to establish a clear temporal relationship with the outcomes. This also raises the possibility of reverse causation, whereby tissue characteristics may have been influenced by disease progression rather than acting solely as predisposing factors. Fifth, the categorization of KMW (0 mm, <2 mm, ≥2 mm) and MT (<2 mm vs. ≥2 mm), and brushing discomfort assessed dichotomously, while clinically relevant, may have led to loss of information, potential misclassification bias, and spurious interaction. Therefore, alternative categorizations or stratification approaches should be explored to better define thresholds for what constitutes inadequate versus adequate KMW, as well as thin versus thick MT, and to clarify their biological relevance in relation to implant-related outcomes and peri-implant diseases. Additionally, KMW was categorized into three groups to explore a potential dose-response relationship and to distinguish the complete absence of KMW from reduced but present KMW; however, this may limit the interpretation of this stratification. Similarly, MT measurements were obtained at a single apico-coronal level at the facial site, which may not fully represent the three-dimensional soft tissue phenotype. Future studies using standardized and reproducible methods of assessment should evaluate different populations, implant characteristics (e.g., macro- and micro-designs), and a wider range of clinical scenarios.

**REFERENCES**

Roccuzzo, A., Imber, J. C., Stähli, A., Romandini, M., Sculean, A., Salvi, G. E., & Roccuzzo, M. (2025). Role of Keratinized Mucosa on the Risk of Peri-Implant Diseases and Soft Tissue Dehiscence in the Posterior Mandible-A 20-Year Prospective Cohort Study. J Periodontal Res, 60(12), 1212-1221. doi:10.1111/jre.70018
